# Validation of a Measure of Loneliness for Homebound Older Adults

**DOI:** 10.1093/geroni/igab046.2373

**Published:** 2021-12-17

**Authors:** Keith Chan, Christina Marsack-Topolewski, Sarah LaFave, Maggie Ratnayake, Jillian Graves, Diane Fenske

**Affiliations:** 1 Hunter College, New York, New York, United States; 2 Eastern Michigan University, Ypsilanti, Michigan, United States; 3 Johns Hopkins University, Baltimore, Maryland, United States; 4 Lori's Hands, Newark, Delaware, United States; 5 Eastern Michigan University, Eastern Michigan University, Michigan, United States

## Abstract

The pandemic has disproportionately impacted older adults, highlighting the need to address social isolation for this population. Homebound older adults are at risk for loneliness, which is a correlate of poor mental and physical health. COVID-19 has exacerbated effects of social isolation by limiting contact with family and other visitors. In-depth empirical validation of loneliness scales is needed to examine the measurement of this construct for homebound older adults who are aging in place. This study examined the reliability and validity of the UCLA Loneliness Scale (v3) for a community-dwelling population of older adults who received home-based support services due to their homebound status or have chronic illness resulting in ADL limitations. Using in-home interviews, data were collected for 175 older adults using the UCLA Loneliness Scale. Reliability and confirmatory factor analyses were conducted to examine its psychometric properties. Findings demonstrated the scale had good internal consistency reliability (ɑ = 0.91). Confirmatory factor analyses indicated a two-factor solution, 1) disconnectedness and 2) connectedness, accounting for 92% of the variability in the 20 items. The lack of meaningful relationships (ƛ = 0.73, p < 0.05) or having someone to turn to (ƛ = 0.68, p < 0.05) substantively contributed to disconnectedness. Feeling that there were people to talk to (ƛ = 0.67, p < 0.05) and turn to (ƛ = 0.76, p < 0.05) contributed to connectedness. Future research can further examine how quality of relationships and benefits of being connected to others can address loneliness and isolation for this population.

